# TAp63 suppresses mammary tumorigenesis through regulation of the Hippo pathway

**DOI:** 10.1038/onc.2016.388

**Published:** 2016-11-21

**Authors:** X Su, M Napoli, H A Abbas, A Venkatanarayan, N H B Bui, C Coarfa, Y J Gi, F Kittrell, P H Gunaratne, D Medina, J M Rosen, F Behbod, E R Flores

**Affiliations:** 1 Department of Molecular Oncology, H. Lee Moffitt Cancer Center, Tampa, FL, USA; 2 Department of Cutaneous Oncology, H. Lee Moffitt Cancer Center, Houston, Tampa, FL, USA; 3 Cancer Biology and Evolution Program, H. Lee Moffitt Cancer Center, Tampa, FL, USA; 4 Department of Molecular and Cellular Biology, Baylor College of Medicine, Houston, TX, USA; 5 Department of Biology and Biochemistry, University of Houston, Houston, TX, USA; 6 Department of Pathology and Laboratory Medicine, University of Kansas Medical Center, Kansas City, KS, USA

## Abstract

Mechanisms regulating the transition of mammary epithelial cells (MECs) to mammary stem cells (MaSCs) and to tumor-initiating cells (TICs) have not been entirely elucidated. The p53 family member, p63, is critical for mammary gland development and contains transactivation domain isoforms, which have tumor-suppressive activities, and the ΔN isoforms, which act as oncogenes. In the clinic, p63 is often used as a diagnostic marker, and further analysis of the function of TAp63 in the mammary gland is critical for improved diagnosis and patient care. Loss of *TAp63* in mice leads to the formation of aggressive metastatic mammary adenocarcinoma at 9–16 months of age. Here we show that TAp63 is crucial for the transition of mammary cancer cells to TICs. When *TAp63* is lost, MECs express embryonic and MaSC signatures and activate the Hippo pathway. These data indicate a crucial role for TAp63 in mammary TICs and provide a mechanism for its role as a tumor- and metastasis-suppressor in breast cancer.

## Introduction

Mammary stem cells (MaSCs) have key roles in the development of breast cancer, in its progression and in the effectiveness of breast cancer therapy. Upstream genes controlling this process are still poorly understood. One critical transcription factor involved in epithelial stem cell maintenance of the mammary gland and skin is the p53 family member and tumor-suppressor gene, *p63*.^1, 2, 3, 4, 5^ *p63* is composed of multiple isoforms with overlapping and unique activities. It is important to note that p63 is used as a diagnostic marker in metaplastic breast cancer with no regard to the existence or activities of p63 isoforms,^6^ and research to date has been focused on the highly expressed isoform, ΔNp63. The p63 isoforms can be placed into two groups: the transactivation domain isoforms, which structurally resemble *p53* and act as tumor suppressors, and the ΔN isoforms, which bind to p53, TAp63 and TAp73 and inhibit their function, thus acting as oncogenes.^7, 8, 9^ In the skin, TAp63 is required to maintain adult dermal stem cells and epidermal progenitor cells, required for wound healing and hair regeneration, in quiescence.^5, 10^ ΔNp63 also has an important role in the skin. Its expression in the basal compartment of the epidermis is required for epidermal stratification and terminal differentiation in the developing and adult skin.^10, 11, 12^ Likewise, the expression patterns of the TAp63 and ΔNp63 isoforms of p63 in distinct mammary progenitor and stem cells suggest different roles for these isoforms in mammary gland development and homeostasis.^13^ Although ΔNp63 is highly expressed in basal cells and is critical for mammary gland development and maturation,^4^ the roles of TAp63 have not been investigated *in vivo* using knockout mouse models. Additionally, mechanisms for TAp63 regulation in MaSCs and how this may impinge on mammary tumorigenesis have yet to be elucidated and are critical for further understanding of how p63 can be used as a diagnostic marker for breast cancer and for therapy.

Recent studies have shed light on functions for the p63 isoforms in breast cancer. TAp63 is not expressed or is present at low levels in high-grade mammary adenocarcinoma, and regulation of microRNA biogenesis through transcriptional regulation of *Dicer* has been implicated in its ability to suppress tumor progression and metastasis.^9, 14^ Other mechanisms for p63's role as a suppressor of tumorigenesis and metastasis have also been shown, including integrin recycling and interactions with transforming growth factor-β.^9, 15, 16^ In mouse models, *TAp63-*deficient mice are highly prone to metastatic mammary adenocarcinoma.^14^ Given the functions of TAp63 in stem cells in the skin, its clear role as a mammary tumor and metastasis suppressor^5, 11, 14^ and the use of p63 as a diagnostic marker in breast cancer, we sought to understand the roles of TAp63 in the regulation of MaSCs and tumor-initiating cells (TICs) using a *TAp63* isoform specific knock out mouse model.^5^

Mechanisms regulating MaSCs and breast cancer stem cells (CSCs) or TICs have not been completely delineated.^17^ For example, aggressive luminal breast cancer subtypes can acquire basal cell and CSC features during their progression,^18^ and basal cell breast cancer may originate from luminal cells.^19^ Recent studies have also revealed that normal breast stem cells and CSCs share some regulatory mechanisms in certain types of breast cancer. For example, coexpression of Sox9 and Slug is sufficient to convert luminal mammary cells into MaSCs capable of mammary gland reconstitution^20^ and tumor development.^21^ Additionally, coexpression of Sox9 and Slug were found to be predictive CSC markers and promoted tumor development and metastasis.^20, 21^ TAZ, a transducer of the Hippo pathway, has been shown to also confer CSC properties onto mammary epithelial cells (MECs) through regulation of genes that regulate cell polarity, such as Scribble (Scrib).^22^ Although it is clear that developmental genes such as p63 regulate MaSCs and the development of breast cancer, the complexity of genes such as p63 with its many isoforms and duplicitous activities in tumorigenesis make it essential to further dissect their functions in the regulation of MaSCs in cancer. Here we examine the roles of the tumor-suppressor gene, *TAp63*, in the development of breast cancer through TICs.

Similar to the p63 isoforms, components of the Hippo pathway have been implicated in breast cancer and progression.^22, 23, 24, 25^ The Hippo pathway functions to regulate cell proliferation, stem cell properties, cell polarity and tumorigenesis.^25^ Some cross-talk between p63 and the Hippo pathway has been discovered recently. ΔNp63 directly interacts with the Hippo effector YAP and is a mediator of YAP function in the epithelium of lung airways.^26^ Additionally, *LKB1*, a transcriptional target of TAp63 in the liver, is mutated in breast cancers^27^ and serves to maintain cell polarity through upstream regulation of Scrib and TAZ, a transducer of the Hippo pathway.^28^ Moreover, TAZ has been shown to confer CSC properties of MECs through regulation of Scrib and cell polarity.^22^ Although connections between cell polarity, the Hippo pathway and the properties of MaSCs have been well established, the upstream regulation of this process by TAp63 was previously not known.

Here we show that the tumor suppressor and transcription factor, *TAp63*, regulates cell polarity and MaSCs through transcriptional regulation of *LKB1* and downstream regulation of Scrib and components of the Hippo pathway. Using genetically engineered mice, human mammary cancer cell lines, orthotopic xenograft mouse models and patient-derived mammary adenocarcinomas, we show that TAp63 serves to regulate the stem cell potential of MEC and TICs. When TAp63 is lost, MECs and mammary cancer cells express embryonic stem cell (ESC) and human normal mammary stem cell (hNMSC) signatures. Loss of *TAp63* in MECs and mammary cancer cells also leads to: (1) loss of cell polarity through the regulation of Scrib and TAZ after serial transplantation, (2) an accumulation of early TICs in serially transplanted mammary glands, and (3) an accumulation of cells with increased tumor-initiating capabilities in tumors derived from the MCF7-shTAp63 orthotopic mammary tumor mouse model. These data reveal novel and crucial roles of TAp63 in the regulation of TICs through the Hippo pathway in the mammary gland and in breast cancer and have important implications for the diagnosis and treatment of breast cancer.

## Results

### Mammary glands from TAp63−/− mice are hyperplastic and have increased regenerative potential

**Figure 1:**
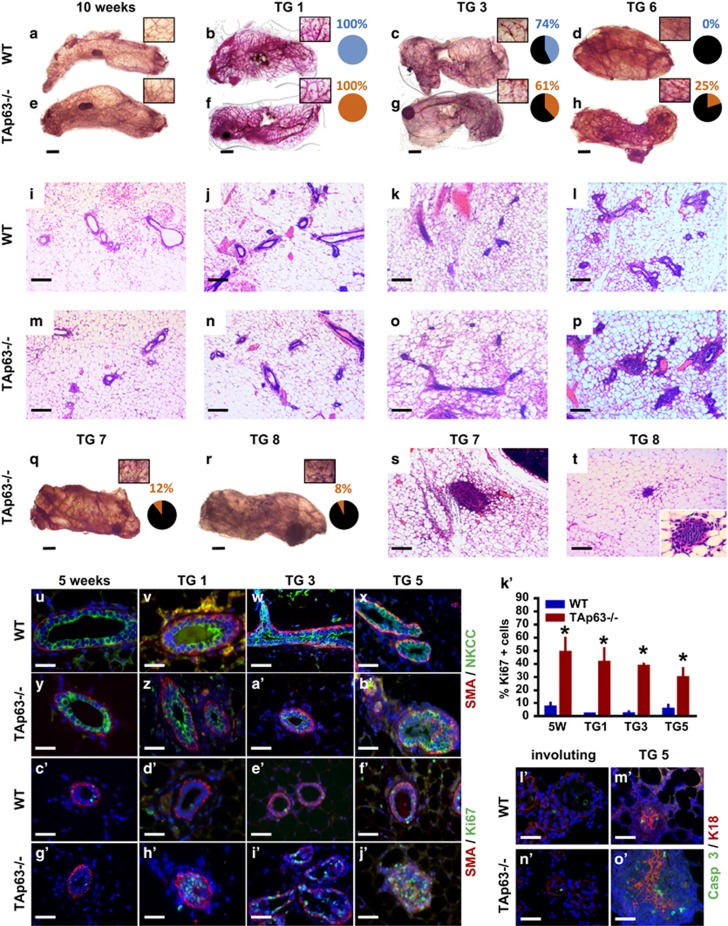
*TAp63−/−* mammary glands are hyperplastic and have increased regenerative potential. (**a**–**h**) Representative whole-mount staining and (**i**–**p**) H&E staining of WT and *TAp63−/−* mammary glands (MG) at 10 weeks of age and subsequent to 1, 3 or 6 serial transplantation passages *in vivo* (TG1, TG3, TG6). (**q** and **r**) Representative whole-mount staining and (**s** and **t**) H&E staining of *TAp63−/−*-transplanted MG at TG7 and TG8. Insets of pie graphs (**a**–**h** and **q**–**r**) indicate the transplanted MG outgrowth coverage of fat pads. For panels (**a**–**t**), *n*=6. Representative micrographs of IF staining of WT and *TAp63−/−* MG at 5 weeks and serially transplanted MG indicated using antibodies for (**u**–**b'**) SMA and NKCC, (**c'**–**j'**) SMA and Ki67. (**k'**) Bar graph showing quantification of Ki67-positive cells from panels (**c'**–**j'**). Asterisks indicate statistical significance, *P*<0.05. (**l'**–**o'**) Representative micrographs of IF staining of WT and *TAp63−/−* MG that are involuting and at TG5 using antibodies for Casp 3 and K18. Scale bar represents 5 mm in panels (**a**–**h**) and (**q**–**r**), 100 μm in (**i**–**p**) and (**s**–**t**), 50 μm in (**u**–**j'**) and (**l'**–**o'**). For panels (**u–o'**), *n*=5.

We have shown previously that *TAp63* is critical for the maintenance of stem cells within the dermis by employing mechanisms that keep these cells in quiescence.^5^ To determine whether TAp63 may similarly regulate stem cells in the mammary gland, we assayed for the regenerative potential of mammary glands from *TAp63−/−* mice. In virgin females at 10 weeks of age, no difference was observed in the development and morphology of mammary glands from wild-type (WT) and *TAp63−/−* mice (Figures 1a and e). However, following serial transplantation, we observed a number of differences between the two genotypes (Figures 1a–t). *TAp63−/−* mammary glands are capable of repopulating WT donor mammary fat pads for eight generations while WT mammary glands only do so for five generations (Figures 1a–h, q and r). The outgrowth coverage, the area of the fat pad repopulated by the MECs, of transplanted WT mammary glands drops from 100% in transplantation generation 1 (TG1) to 0% in generation 6 (TG6) (Figures 1b–d; Supplementary Table S1). In contrast, the outgrowth coverage of *TAp63−/−* mammary glands is 25%±32% in TG6, 12%±27% in TG7 and 8%±11% in TG8 (Figures 1h, q and r; Supplementary Table S1). The outgrowth rate, calculated by dividing the number of fat pads with mammary outgrowth coverage by the total number of mammary fat pads inoculated with MECs, of transplanted *TAp63−/−* mammary glands is significantly higher than WT mammary glands from TG5 to TG8 (Supplementary Figure S1). *TAp63−/−* mice had 83% mammary outgrowth coverage compared with 58% in WT mice at TG5. Although WT mice had no outgrowth coverage at TG6, TG7 and TG8, *TAp63−/−* mice had outgrowth coverages of 58, 36 and 62% for each of these transplant generations, respectively (Supplementary Figure S1). Additionally, the morphology of *TAp63−/−* mammary glands differed from that of WT-transplanted glands (Figures 1i–p, s and t). The *TAp63−/−* mammary glands lacked lumens, and these areas were filled with cells (Figures 1o, p, s and t). Detailed analyses using immunofluorescence (IF) staining for the basal marker, smooth muscle actin (SMA) and the luminal marker, Na-K-Cl cotransporter (NKCC), revealed that the basal and luminal layers of the mammary gland are highly disorganized in *TAp63−/−* glands. This disorganization was apparent as early as the first transplantation (TG1), with severe defects noted by TG5 (Figures 1u–b'). In particular, we found that basal cells detached from the basement membrane and localized to the luminal cell compartment (Figures 1a' and b'). We asked whether the *TAp63−/−* mammary glands are hyperplastic by immunostaining with an antibody for Ki67 (Figures 1c'–j'). Indeed, we found an increase in Ki67-positive cells within the lumen of the mammary glands of 5-week-old *TAp63−/−* virgin females (50%±10%) compared with WT females of the same age (5%±3% Figures 1g' and k'). We also found increased Ki67-positive cells in *TAp63−/−* mammary glands after each transplantation (Figures 1h'–k'). The *TAp63−/−* TG1, TG3 and TG5 glands contained 40%±12%, 40%±2% and 35%±5%, respectively, while the WT-transplanted glands had 3%±5% for each passage (Figure 1k'), indicating an increase in proliferating cells within the mammary gland of *TAp63−/−* mice even in virgin females.

Apoptosis is a critical step in lumen formation of the mammary gland and in mammary gland involution after pregnancy and weaning. We therefore asked whether the *TAp63−/−* mammary glands have defects in apoptosis. We found cleaved caspase 3-positive cells within WT and *TAp63−/−* involuting mammary glands in addition to positive cells within the disorganized *TAp63−/−* TG5 mammary glands. The number of cleaved caspase 3-positive cells was comparable to what is detected in WT TG5 mammary glands, indicating that *TAp63−/−* mammary glands do not have defects in the induction of apoptosis (Figures 1l'–o'). The apoptotic cells in *TAp63−/−* mammary glands appear to be mislocalized in TG5 glands (Figure 1o').

### Transplanted TAp63−/− mammary glands have defects in cell polarity

**Figure 2:**
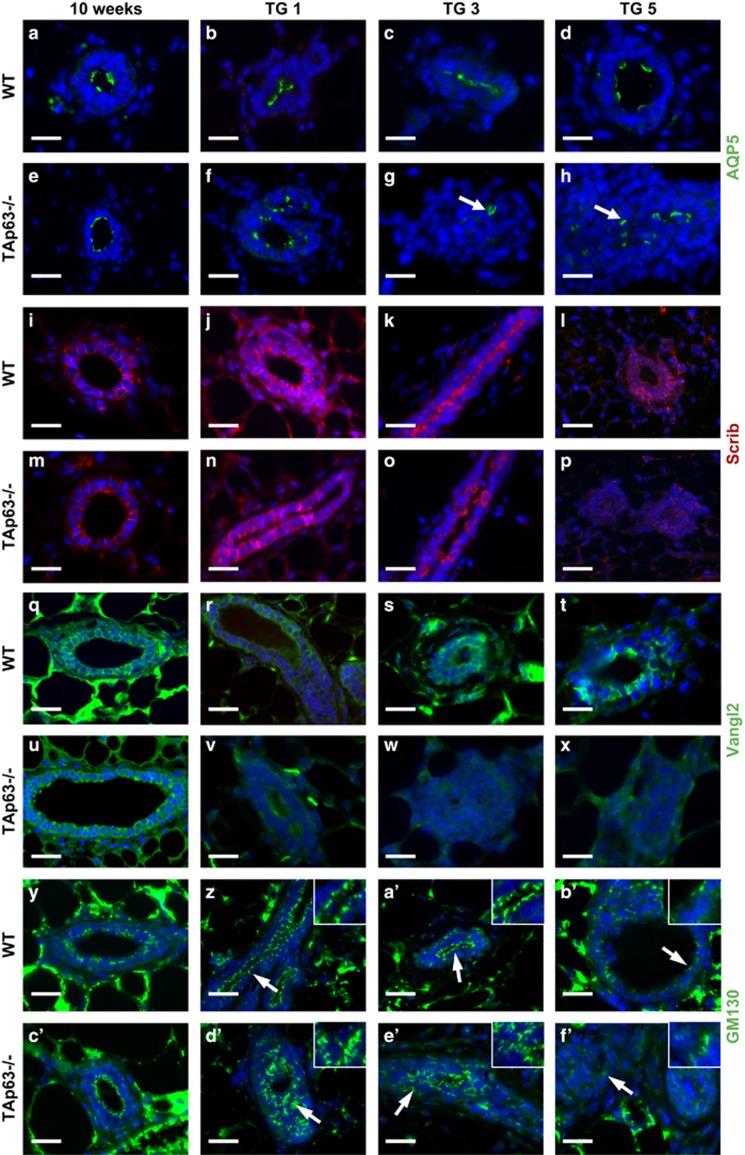
*TAp63−/−* mammary glands have cell polarity defects after transplantation. Representative IF micrographs of WT and *TAp63−/−* mammary glands at 10 weeks of age and subsequent to 1, 3, or 5 serial transplantation passages *in vivo* (TG1, TG3, TG5) using antibodies for: (**a**–**h**) APQ5, (**i**–**p**) Scrib, (**q**–**x**) Vangl2 and (**y**–**f'**) GM130. Arrows indicate positive cells in panels (**g**–**h**) and areas further magnified in insets in the upper right corner of panels (**z–b'**) and (**d'**–**f'**). Scale bar represents 50 μm in panels (**a**–**f'**). *n*=5.

Cell polarity are common pathways altered in human breast cancer, which are also associated with cellular hyperproliferation and disorganization of the mammary gland structure.^27, 29, 30, 31^ To determine whether these pathways are deregulated in the absence of *TAp63* leading to the observed solid gland structure and hyperplastic phenotype of *TAp63−/−*transplanted glands, we performed IF staining for markers of cell polarity at 10 weeks after birth in virgin females and after 1, 3 and 5 rounds of transplantation (TG1, TG3 and TG5). Both WT and *TAp63−/−* luminal epithelial cells express AQP5, an apical marker of luminal cells, on one side of the cells indicating no defects in apical–basal cell polarity in the absence of *TAp63* (Figures 2a–h). With later transplantation passages (TG3 and TG5), the AQP5-positive cells within the *TAp63−/−* mammary glands are disorganized as indicted by the arrows, AQP5 is still expressed on one side of the cell (Figures 2g and h) in some cells, but most TAp63*−/−* cells lost apical–basal cell polarity during transplantation (compare Figures 2e–h). In contrast, the AQP5-positive cells within the WT mammary gland line up on the apical side to form the lumen (Figures 2c and d). These data suggest that the *TAp63−/−* luminal MECs have defects in cell polarity. Scrib and Vangl2 serve to maintain cell polarity.^29, 32, 33^ To ask whether the *TAp63−/−* mammary glands have defects in cell polarity, we performed IF staining for Scrib and Vangl2. Although Scrib and Vangl2 were expressed similarly in 10-week old WT and *TAp63−/−* mammary glands from virgin females, we found that these proteins are mislocalized or decreased in expression in *TAp63−/−*-transplanted glands (Figures 2i–x). These data indicate that *TAp63−/−* mammary glands have defects in cell polarity. Scrib has also been shown to control polarization of the Golgi and is required for the establishment of cell polarity.^31^ We therefore used a marker to label the Golgi (GM130) and found that transplanted *TAp63−/−* glands as early as passage 1 had mislocalized staining of GM130, further indicating defects in cell polarity and suggesting regulation of Scrib by TAp63 in the mammary gland (Figures 2y–f').

### TAp63−/− mammary glands acquire an increased number of early TICs through the activation of an epithelial–mesenchymal transition (EMT) program

**Figure 3:**
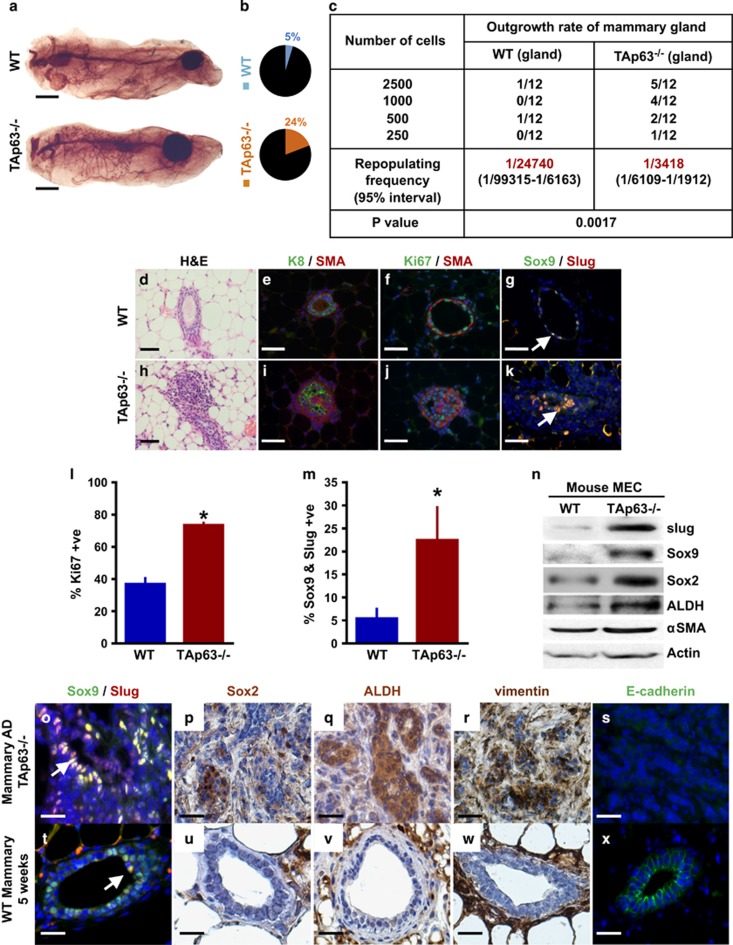
*TAp63−/−* mammary glands have increased numbers of TICs. (**a**) Representative whole-mount staining of MG generated from WT and *TAp63−/−*MECs in limiting dilution assay (LDA). Scale bar represents 50 mm. (**b**) Pie charts indicating MG outgrowth rate from LDA. Colored pie slices with numbers indicate outgrowth coverage. (**c**) Table indicating MG outgrowth of LDA using MECs from WT and *TAp63−/−* mammary glands from panel (**a**). (**d**–**k**) H&E staining (**d** and **h**) or IF staining (**e**–**g** and **i**–**k**) of outgrown MG from LDA using: keratin 8 (K8; green), SMA (red), Ki67 (green), Sox9 (green), and slug (red). Scale bar represents 50 μm in panels (**d**–**k**). (**l**) Bar graph showing quantification of Ki67-positive cells from panels (**f**) and (**j**). (**m**) Bar graph showing quantification of Sox9 and Slug double-positive cells from panels (**g**) and (**k**). Asterisk represents statistical significance, *P*<0.05. (**n**) Representative western blotting analysis of WT and *TAp63−/−* mouse MECs using antibodies for slug, Sox9, Sox2, ALDH and αSMA. Actin was used as a loading control. (**o**–**x**) IF or IHC staining of mammary adenocarcinomas from *TAp63−/−* mice (**o**–**s**) or MG from 5-week-old WT mice (**t**–**x**) using antibodies for Sox9 (green), slug (red), Sox2 (brown), ALDH (brown), vimentin (brown) and E–cadherin (green). DAPI (blue) or hematoxylin (purple) were used as counterstains. Arrow indicates example of double-positive nuclei (yellow). Scale bar represents 50 μm in panels (**o**–**s**). All scale bars represent 50 μm. *n*=6 for all experiments.

The *TAp63−/−* mammary glands have increased regenerative potential as assessed by an increased capacity to transplant these mammary glands into syngeneic mice, suggesting that the *TAp63−/−* mammary glands have an increased number of TICs. To determine whether *TAp63−/−* mammary glands contain a greater number of TICs than WT mammary glands, we performed limiting dilution assays. These assays were performed by isolating single MECs from WT and *TAp63−/−* mouse mammary glands and injecting 250, 500, 1000, and 2500 cells into the cleared fat pads of syngeneic WT mice. We found that *TAp63−/−* MECs had a greater outgrowth rate and outgrowth coverage than WT MECs (Figures 3a–c and Supplementary Figure S2). We found the outgrowth rate from *TAp63−/−* MECs, calculated by dividing the number of fat pads with mammary outgrowth by the total number of mammary fat pads inoculated with MECs, to be five times greater than that of MECs derived from WT mice (Figures 3b and c). We also calculated the amount of outgrowth coverage by calculating the area of the fat pad repopulated by the MECs. Using this calculation, we found that *TAp63−/−* MECs have an outgrowth coverage that is 10 times greater than that of WT MECs (Supplementary Figure S2). By histological analysis of these glands, we found that *TAp63−/−* MECs formed disorganized mammary glands lacking a luminal space (compare Figures 3d and h) as we had previously noted after serial transplantation (Figure 1). We also found the *TAp63−/−* mammary glands from the limiting dilution experiment to be highly disorganized by analyzing well-characterized markers of the mammary gland (Figures 3h–k). By IF staining for the luminal marker, keratin 8, and the basal marker, SMA, we found basal cells in *TAp63−/−* mammary glands to be mislocalized to the luminal compartment (compare Figures 3e and i). Additionally, the *TAp63−/−* mammary glands had an increase in the number and location of proliferative cells as assessed by Ki67 staining (Figures 3f and j). *TAp63−/−* mammary glands had 75%±1% Ki67-positive cells compared with 38%±2% in WT mammary glands (Figure 3l). Taken together, these results recapitulate the defects detected after serial transplantation of *TAp63−/−* mammary glands and suggest that these mammary glands are hyperplastic and may be enriched for early TICs.

Sox9 and Slug double-positive cells have been shown to mark MaSCs, TICs and CSCs.^20, 21^ Further, Sox9 and Slug activate an EMT program in MECs maintaining them in the stem cell stage and have been implicated as markers of MaSCs, TICs and CSCs based on this activity.^20, 21^ To ask whether *TAp63−/−* mammary glands formed from the limiting dilution experiment have increased number of MaSCs, TICs and CSCs, we performed IF staining for Sox9 and Slug (Figures 3g and k). Indeed, we found that *TAp63−/−* mammary glands from limiting dilution experiments had 22%±8% Sox9 and Slug double-positive cells compared with just 5%±2% in WT mammary glands from limiting dilution assays (Figures 3g, k and m), indicating that these mammary glands are enriched for early TICs. We also performed western blotting analysis using MECs from 10-week-old virgin WT and *TAp63−/−* female mice and found that both Sox9 and Slug are expressed at much higher levels in *TAp63−/−* versus WT MECs (Figure 3n). Further, we analyzed additional markers for TICs, Sox2 and ALDH1 and found these to be highly expressed in *TAp63−/−* MECs, suggesting an enrichment for early TICs in *TAp63−/−* mammary glands (Figure 3n). Finally, we found the mesenchymal marker, SMA, present at higher levels in MECs extracted from *TAp63−/−* compared with WT mammary glands (Figure 3n), in agreement with previous data showing the association of EMT with an acquisition of early TIC characteristics.^20, 21^ We further demonstrated that *TAp63−/−* mammary adenocarcinomas express the luminal marker NKCC1, are ER positive and lose expression of the polarity protein, Scrib (Supplementary Figures S3a–c). Given the expansion of early TICs in transplanted *TAp63−/−* mammary glands, we asked whether this population was expanded in *TAp63−/−* mammary adenocarcinomas. Indeed, we found an enrichment of TICs in mammary adenocarcinomas from *TAp63−/−* mice by IF using markers for MaSCs (Slug, Sox9, Sox2 and ALDH) (Figures 3o–q and t–v and Supplementary Figures S6a–r and y–a') and an upregulation of the mesenchymal marker, vimentin (Figures 3r and w and Supplementary Figures S6s–x and b'), with a concomitant downregulation of the epithelial marker, E-cadherin (Figures 3s–x), demonstrating EMT in these tumors and suggesting that cells characterized are TICs. Mammary glands from 5-week-old WT mice were used as controls (Figures 3t–x). Taken together, our data indicate that TAp63 serves to maintain MECs. When *TAp63* is lost, MECs undergo EMT and transition to acquire characteristics of TICs.

### TAp63−/− MECs are significantly enriched in MaSC and ESC signatures

**Figure 4:**
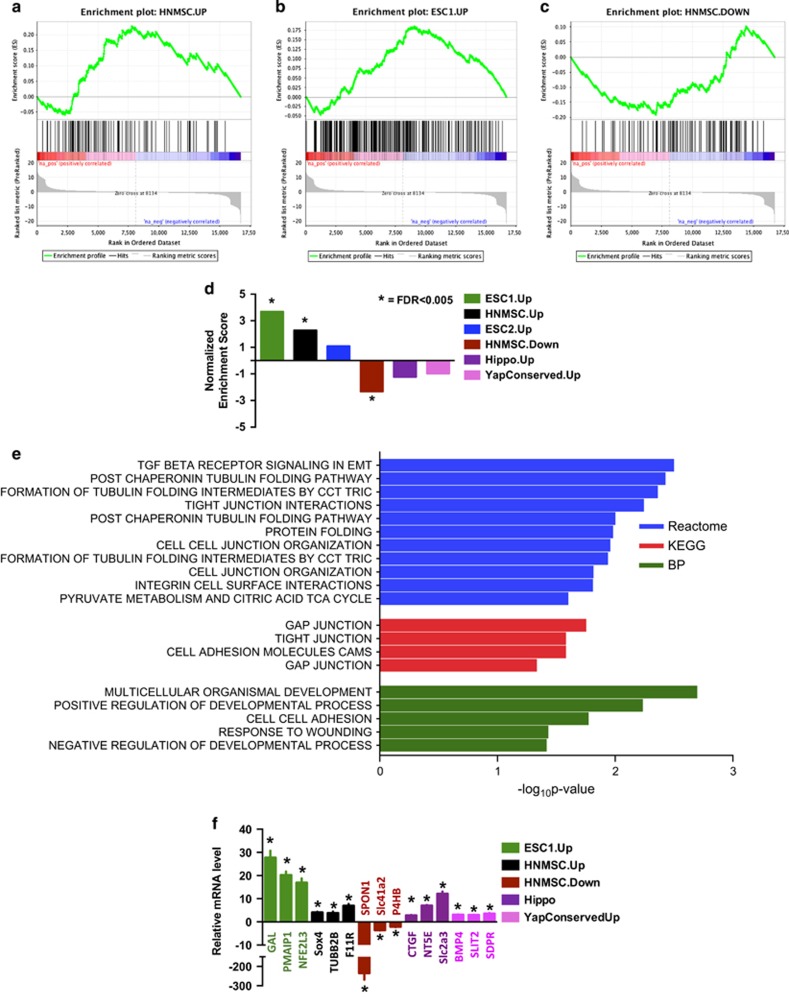
*TAp63−/−* MECs are significantly enriched in hNMSC and ESC signatures. Gene set enrichment analyses of upregulated genes in hNMSCs (**a**) and ESCs (**b**) reveals positive enrichment of *TAp63−/−* MECs, while downregulated genes of hNMSCs (**c**) shows negative enrichment of *TAp63−/−* MECs. (**d**) Normalized enrichment scores for hNMSC, ESC, Hippo and Yap pathway signatures in *TAp63−/−* MECs. (**e**) Pathway enrichment analysis reveals enrichment of tubulin, tight and gap junction and epithelial-to-mesenchymal transition pathways as analyzed from BP, KEGG and Reactome databases. (**f**) qRT-PCR validation of targets that are common to *TAp63−/−* MECs for each of the signatures.

To determine critical pathways present in *TAp63−/−* MECs, we performed RNA-seq analysis using RNA extracted from WT and *TAp63−/−* MECs. Using Gene Set Enrichment Analysis, we found that *TAp63−/−* MECs are significantly enriched in hNMSC and ESC signatures (*q*<0.0001)^22, 28, 34, 35^ (Supplementary Table S2 and Figures 4a–d). Using pathway analysis for genes that are common in *TAp63−/−* MECs and hNMSC and ESC gene signatures, we found a significant enrichment for pathways involved in tubulin folding, cell adhesion and tight and gap junctions, all pathways influencing cellular polarity and affecting stem cell potential^22, 36, 37^(Figure 4e).

We then performed quantitative real-time PCR (qRT-PCR) in order to validate genes that are significantly enriched in the hNMSC and ESC signatures. As shown in Figure 4e, *GAL*, *PMAIP1* and *NFE2L3*, which were previously reported to be upregulated in the ESC1 signature,^34^ were also upregulated in *TAp63−/−* MECs. Genes enriched in the hNMSC signature were also validated; we found *Sox4*, *TUBB2B* and *F11R* to be upregulated and *SPON1*, *Slc41a2* and *P4HB* to be downregulated in *TAp63−/−* MECs (Figure 4f) similar to the expression levels found in hNMSCs.^35^ These data indicate that *TAp63−/−* MECs express a transcriptional profile resembling that of both embryonic and normal MaSCs and suggest that MaSCs accumulate in *TAp63*-deficient mammary glands through transcriptional regulation of these stem cell pathways. The Hippo pathway has also been implicated in conferring stem cell properties in multiple tissues,^38, 39^ and we found the Hippo pathway to be deregulated in *TAp63−/−* MECs (Figure 4d). Therefore, we also assessed the expression of genes in the published Hippo- and YAP-conserved signatures.^22, 28^ We found that several of these mRNAs, including *CTGF*, *NT5E*, *Slc2a3*, *BMP4*, *SLIT2* and *SPDR*, were upregulated in *TAp63−/−* MECs, suggesting regulation of the Hippo pathway by TAp63.

### TAp63 regulates the Hippo pathway

**Figure 5:**
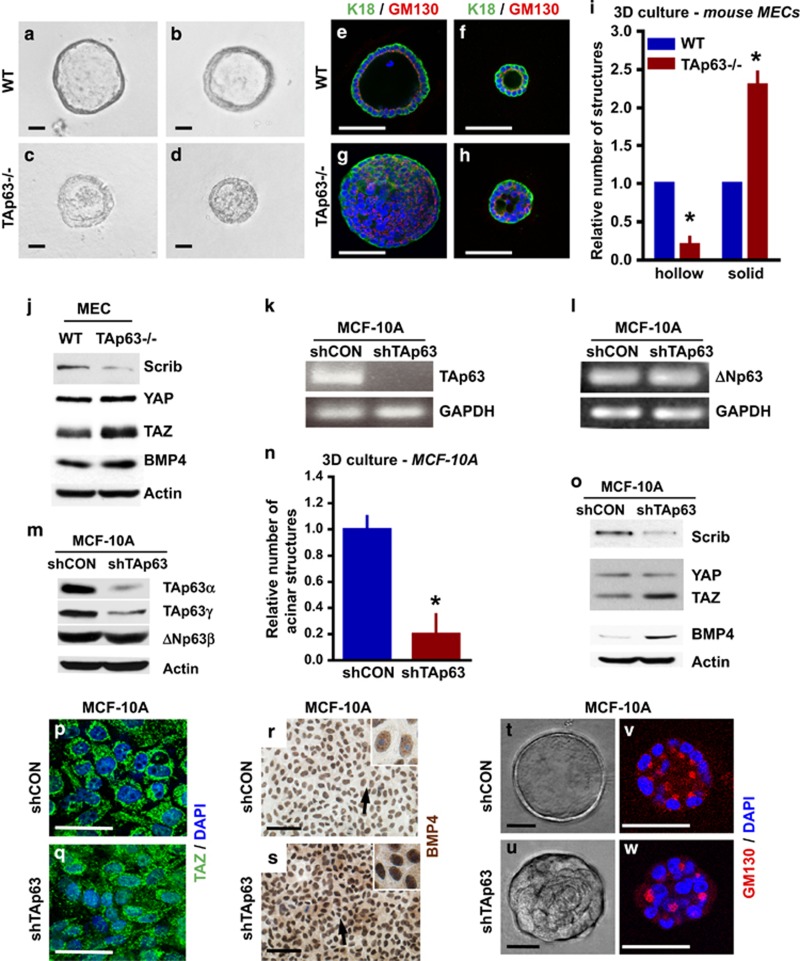
TAp63 regulates mammary gland cell polarity through the Hippo pathway. (**a**–**d**) Bright-field micrographs of primary mouse MECs grown in 3D cultures. Acinar structures from WT MECs grown in 3D culture (**a** and **b**) and solid structures from *TAp63−/−* MECs grown in 3D culture (**c** and **d**). (**e**–**h**) Representative confocal micrographs of WT and *TAp63−/−* 3D cultures immunostained with K18 (green) and GM130 (red). DAPI (blue) was used as a counterstain. (**i**) Bar graph showing the relative formation rate of hollow and solid acinar structures from a total of 268 WT and 207 of *TAp63−/−* 3D structures. Asterisks indicate statistical significance, *P*<0.05. (**j**) Representative western blotting (WB) of MECs of the indicated genotypes using antibodies against Scrib, YAP, TAZ and BMP4. Actin was used as a loading control. (**k**–**l**) Semiquantitative RT-PCR for *TAp63* (**k**) and *ΔNp63* (**l**) in MCF10A-shRNA specific for TAp63 and a non-specific control shCON. (**m**) Representative WB of MCF10A-shCON or -shTAp63 using antibodies for TAp63 and ΔNp63. Actin was used as a loading control. (**n**) Bar graph showing relative acinar formation of MCF10A-shCON or -shTAp63 cells. Six hundred structures were counted for each genotype. Asterisks show statistical significance, *P*<0.05. (**o**) Representative WB of MCF10A-shCON or -shTAp63 using antibodies for Scrib, YAP, TAZ and BMP4. (**p** and **q**) Representative confocal micrographs of MCF10A-shCON cells (**p**) or -shTAp63 (**q**) and immunostained with TAZ (green). DAPI (blue) was used as a counterstain. (**r** and **s**) IHC staining of MCF10A-shCON or -shTAp63 cells using an antibody for BMP4 (brown). Hematoxylin (purple) was used as a counterstain. (**t** and **u**) Representative bright-field micrographs of MCF10A-shCON or -shTAp63 in 3D culture. (**v** and **w**) Representative confocal micrographs of MCF10A-shCON (**v**) or -shTAp63 (**w**) and immunostained GM130 (red). DAPI (blue) was used as a counterstain. All scale bar represents 50 μm. *n*=3 for all experiments. GAPDH, glyceraldehyde 3-phosphate dehydrogenase.

To understand the mechanism employed by TAp63 to acquire characteristics of TICs and cell polarity, we cultured WT and *TAp63−/−* mouse MECs in three-dimensional (3D) culture (Figures 5a–h). *TAp63−/−* 3D cultures formed more solid (85%) than hollow (15%) acinar structures compared with 67% solid and 43% hollow acini in WT cultures (Figure 5i). To determine whether *TAp63−/−* mammary acini had defects in cell polarity, as we had detected in transplanted *TAp63−/−* mammary glands, we performed IF staining using the polarity and Golgi marker, GM130. Although WT acini had normal polarity (Figures 5e and f), *TAp63−/−* acini contained random GM130 staining and were highly disorganized (Figures 5g and h). This result recapitulates the polarity defects noted in the transplanted *TAp63−/−* mammary glands.

To further understand whether polarity is regulated through Scrib, as our previous data suggested (Figures 2i–x), we performed western blotting analysis and found that Scrib is present at low levels in *TAp63−/−* MECs (Figure 5j). Because Scrib is upstream of the Hippo pathway, we asked whether YAP and TAZ are increased and whether their downstream target, bone morphogenetic protein 4 (BMP4), of this pathway is activated. We found that TAZ but not YAP levels are increased in *TAp63−/−* MECs and that their downstream target, BMP4, is also increased, suggesting that the Hippo pathway is activated in MECs deficient for *TAp63* (Figure 5j).

### The Hippo pathway is activated after TAp63 knockdown in MCF10A cells

To further determine whether the Hippo pathway is activated by TAp63, we knocked down TAp63 in human MECs, MCF10A cells (Figures 5k–m and Supplementary Figure S4). This resulted in downregulation of TAp63 mRNA and protein and no change in the expression of ΔNp63 (Figures 5k–m). As seen in the *TAp63−/−* MECs, MCF10A cells expressing the short hairpin RNA (shRNA) for TAp63 had fewer hollow acini (Figure 5n) and a decrease in Scrib expression with downstream activation of TAZ and BMP4 (Figure 5o). We further demonstrated using confocal imaging that TAZ localized to the nucleus of MCF10A cells deficient for TAp63 (Figures 5p and q) consistent with the activation of TAZ. Finally, the downstream target of TAZ, BMP4 was upregulated in MCF10A cells deficient for TAp63 (Figures 5r and s). 3D cultures of MCF10A cells deficient for TAp63 exhibited a loss of cell polarity as assessed by GM130 IF staining (Figures 5t–w). These data further suggest that TAp63 regulates cell polarity through the Hippo pathway.

### TAp63 activates the Hippo pathway through transcriptional regulation of LKB1

**Figure 6:**
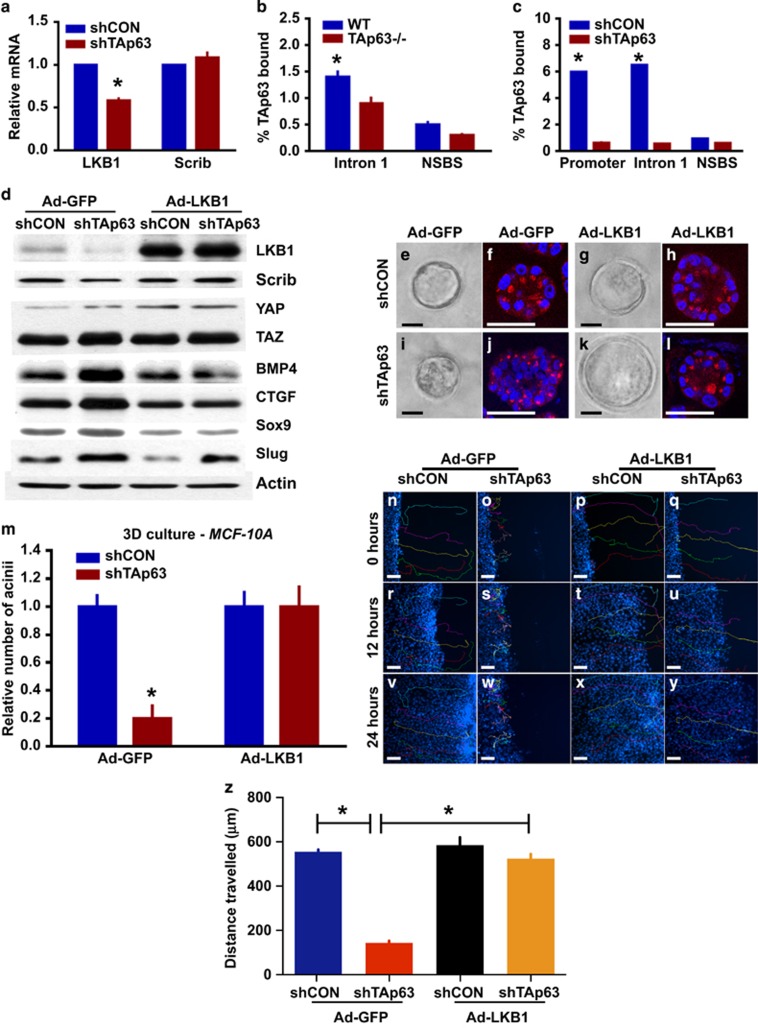
TAp63 regulates cell polarity and the Hippo pathway through transcriptional regulation of LKB1. (**a**) qRT-PCR for Scrib and LKB1 in MCF10A-shTAp63 or -shCON cells. Asterisks show statistical significance, *P*<0.05. (**b** and **c**) qRT-PCR of chromatin immunoprecipitation assay indicating the percentage of TAp63 bound to the *LKB1* promoter in WT and *TAp63−/−* mouse MECs (**b**) and MCF10A-shCON or -shTAp63 cells (**c**). NSBS indicates non-specific binding site. Asterisks show statistical significance, *P*<0.05. (**d**) Representative western blotting analysis of MCF10A-shCON or -shTAp63 cells and transduced with Ad-GFP or Ad-LKB1 using antibodies for LKB1, Scrib, YAP, TAZ, BMP4, CTGF, Sox9 and Slug. Actin was used as a loading control. (**e**–**l**) Bright-field micrographs (**e**, **g**, **l**, **k**) and fluorescent micrographs (**f**, **h**, **j**, **l**) of MCF10A-shCON or -shTAp63 cells and transduced with Ad-GFP or Ad-LKB1 using antibodies for GM130 (red). DAPI (blue) was used as a counterstain. (**m**) Bar graph showing relative acinar formation in 3D cultures of MCF10A-shCON and -shTAp63 cells and MCF10A-shCON and -shTAp63 transduced with Ad-GFP or Ad-Lkb1. Six hundred structures were counted for each condition. Asterisks show statistical significance, *P*<0.05. (**n**–**y**) Frame-by-frame *in vivo* tracking of cell movement within 24 h after wounding. (**z**) Bar graph showing the average distance travelled by 20 cells within 24 h after wounding. Asterisks show statistical significance, *P*<0.05. All scale bars represent 50 μm. *n*=3 for all experiments.

LKB1 is upstream of the MARK kinase family members (MARK1, MARK2 and MARK4), which controls Scrib expression and localization to regulate the Hippo pathway.^28^ We have shown previously that *Lkb1* is transcriptionally activated by TAp63 to regulate glucose metabolism.^40^ To determine whether *Scribble* or *Lkb1* are transcriptional targets of TAp63 in MECs, we assessed their mRNA levels in MCF10A and MCF10A cells expressing an shRNA for *TAp63* (Figure 6a). We found the levels of *Scrib* mRNA to be unchanged in cell lines deficient for *TAp63*, but *LKB1* mRNA was present at significantly lower levels in the absence of TAp63, suggesting that TAp63 may be transcriptionally regulating *LKB1* in MECs (Figure 6a). We therefore performed a chromatin immunoprecipitation analysis using an antibody for TAp63 and the *LKB1* promoter in mouse and human MECs deficient for *TAp63*. We identified two sites within the *LKB1* promoter and within intron 1 where TAp63 binds (Supplementary Table S3), indicating that TAp63 transcriptionally activates *LKB1* in MECs as it did in other tissues (Figures 6b and c).^40^

### Re-expression of LKB1 rescues cell polarity and stem cell defects of TAp63-deficient MECs

We performed immunoblotting on MCF10A and MCF10A cells expressing shTAp63 and found that MCF10A cells deficient for TAp63 expressed low levels of LKB1 and Scrib and increased levels of TAZ, indicating activation of the Hippo pathway subsequent to deletion of *TAp63* (Figure 6d). Re-expression of LKB-1 in *TAp63*-deficient MCF10A cells inactivated the Hippo pathway as indicated by downregulation of BMP4 and connective tissue growth factor (CTGF) (Figure 6d and Supplementary Figure S4), indicating that TAp63 activates the Hippo pathway through transcriptional activation of *LKB1*. TAZ has been found to confer CSC-related traits onto MECs,^22^ indicating that loss of *TAp63* may likewise confer these traits through activation of TAZ. We found that re-expression of LKB-1 in *TAp63*-deficient MCF10A resulted in a downregulation of markers associated with CSC, TAZ, Slug and Sox9 (Figure 6d). To determine whether transcriptional regulation of *LKB1* by TAp63 is causal in the cell polarity defects in *TAp63*-deficient MCF10A cells, we performed assays in 3D and 2D tissue culture to assess cell polarity before and after the forced expression of LKB1 in *TAp63*-deficient MCF10A cells (Figures 6e–z). In 3D cultures, *TAp63*-deficient MCF10A cells have fewer acinar structures and the polarity marker, GM130, is expressed randomly (Figures 6e–l). Re-expression of LKB1 in *TAp63*-deficient MCF10A cells rescued the acinar structures (Figure 6m) and polarity defect (compare Figures 6j and l). We also performed wound-healing assays in 2D cultures to assess polarity using live imaging (Figures 6n–z and Supplementary Figures S5a and b). We found that *TAp63*-deficient MCF10A cells exhibited a delay in wound closure owing to loss of cell polarity (Figures 6e–z and Supplementary Figure S5). Re-expression of LKB1 in *TAp63*-deficient MCF10A cells reinstall cell polarity (Figures 6e–m) and appropriate wound closure (Figures 6n–z and Supplementary Figures S5c and d), indicating that activation of LKB1 and the downstream regulation of the Hippo pathway by TAp63 are critical for maintenance of cell polarity in MECs. Taken together, our data indicate that TAp63 maintains TICs through the maintenance of cell polarity by the transcriptional regulation of *LKB1* and the downstream regulation of the Hippo pathway and suggest this as a possible mechanism for its action as a tumor and metastasis suppressor.

### TAp63 loss unleashes the tumor-initiating potential of human mammary cancer cells

**Figure 7:**
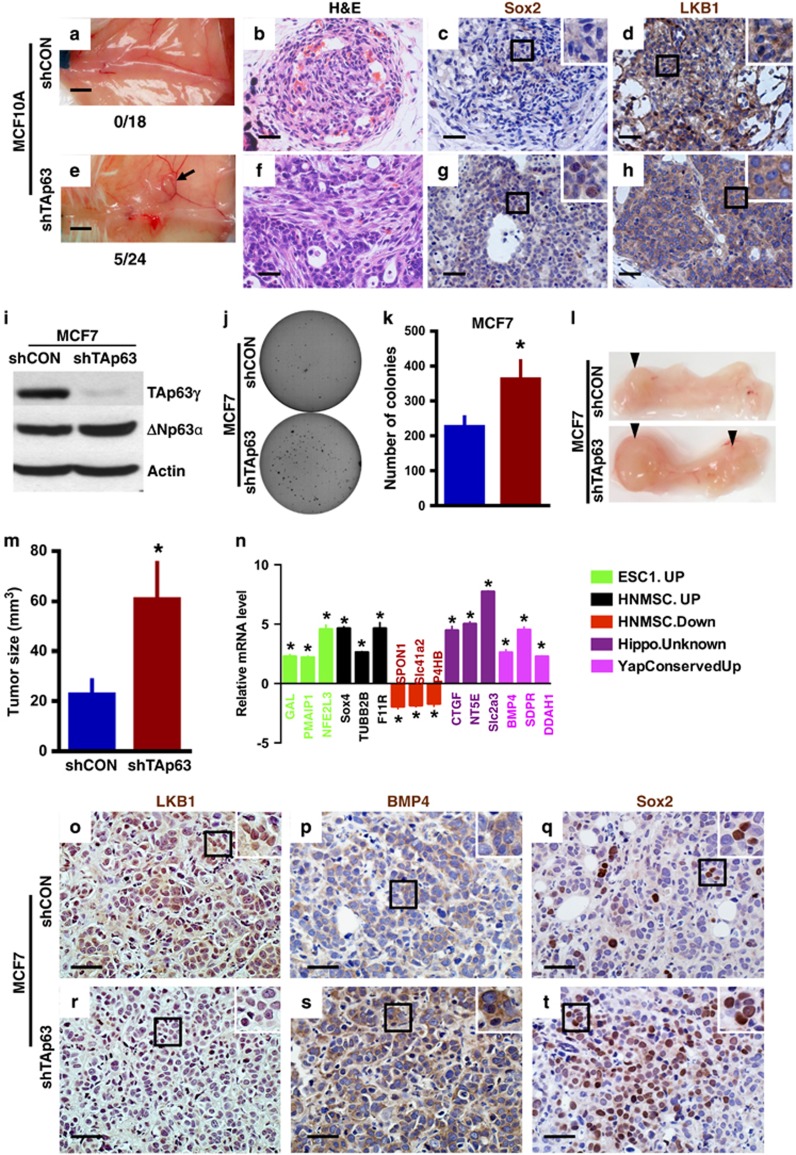
Enrichment of TICs in MCF10A and MCF7 tumors lacking TAp63. (**a** and **e**) Representative micrographs of orthotopic xenograft mouse tumors from mice injected with MCF10A-shCON and -shTAp63. Numbers under micrographs indicate the number of glands with tumor over the total injected glands. Black arrow indicates tumor. (**b** and **f**) Representative H&E and immunostaining of mammary tumors derived from MCF10A-shCON and -shTAp63 orthotopic xenograft mice using antibodies for Sox2 (brown) (**c** and **g**) and Lkb1 (brown) (**d** and **h**). Hematoxylin (purple) was used as a counterstain. Black squares indicate areas further magnified in insets in the upper right corner of each panel. All scale bars represent 50 μm. For panels (**a**–**h**), *n*=6. (**i**) Representative western blotting analysis of MCF7-shCON or -shTAp63 and using antibodies for TAp63 and ΔNp63. Actin was used as a loading control. (**j**) Representative micrographs of MCF7-shCON and -shTAp63 cells cultured in soft agar. (**k**) Bar graph showing the quantification of colonies in soft agar generated from the experiment in panel (**j**). Asterisks show statistical significance, *P*<0.05. For panels (**i**–**k**), *n*=6. (**l**) Representative photographs of orthotopic xenograft mouse tumors from mice injected with MCF7-shCON and -shTAp63. (**m**) Average volume of xenograft mouse tumors from mice injected with MCF7-shCON and -shTAp63 cells. (**n**) qRT-PCR for the indicated mRNAs from mammary tumors derived from MCF7-shTAp63 versus -shCON orthotopic xenograft mice. Data are mean±s.d., *n*=3, *versus MCF7-shCON cells, *P*<0.01, two-tailed *t*-test. (**o**–**t**) Representative IHC staining of mammary tumors derived from MCF7-shCON and -shTAp63 orthotopic xenograft mice. For panels (**l**, **m** and **o**–**t**), *n*=6.

We have shown previously that loss of *TAp63* results in metastatic mammary adenocarcinoma in mouse models (Figures 3o–s).^14^ We have further shown that *TAp63*-deficient tumors are enriched in early TICs (Figures 3n–q). Additionally, we have found that aggressive human mammary adenocarcinomas lose the expression of TAp63.^14^ To further demonstrate that *TAp63* is a tumor suppressor in human mammary adenocarcinoma and regulate the tumor-initiating potential of mammary cancer cells, we knocked down *TAp63* in the non-tumorigenic MEC lines, MCF10A, and investigated the tumorigenic potential using an orthotopic xenograft mouse model. Twenty-one percent of MCF10A-shTAp63 glands formed tumors (5 out of a total of the 24 injected glands) while no tumors formed in mice injected with MCF10A-shCON (0 out of a total of the 18 injected glands; Figures 7a, b, e and f). These data indicate the tumor-initiating potential of loss of TAp63 given the non-tumorigenic nature of MCF10A cells. Further immunohistochemical (IHC) staining revealed that MCF10A-shTAp63 tumors contained a higher level of Sox2-positive cells (Figures 7c and g), indicating an enrichment in TICs in these tumors. To future explore the mechanism of TAp63 in TICs, MCF7 mammary adenocarcinoma cells were used. MCF7 cells formed colonies in soft agar, MCF7 cells lacking TAp63 formed almost two times more colonies in soft agar (Figures 7i–k). We also assayed the tumorigenic potential of MCF7 cells deficient for *TAp63* using an orthotopic xenograft mouse model. Tumors arising from MCF7-shTAp63 cells were three times bigger in volume then MCF7-shCON cells (Figures 7l and m), indicating that loss of TAp63 provides a growth advantage of MCF7 mammary cancer cells. To determine whether TAp63 is regulating the stem cell potential in tumors derived from the MCF7 orthotopic mammary tumor model, we assessed the expression of gene signatures in the ESC, hnMSC and Hippo pathways as we showed in the MECs in Figure 4f. We found that MCF7-shTAp63 tumors were enriched for these signatures as we had shown in *TAp63−/−* MECs (Figure 7n). We also assessed the expression of the TAp63 target, LKB1, in MCF7-shTAp63 as well as MCF10A-shTAp63 tumors and found that the expression was decreased in these tumors compared with those derived from MCF10A-shCON or MCF7-shCON cells (Figures 7d, h, o and r). Concomitantly, we found that the downstream Hippo target BMP4 was upregulated in MCF7-shTAp63 tumors (Figures 7p and s). Finally, in agreement with our previous data, the CSC marker, Sox2, was highly upregulated in MCF7-shTAp63 tumors compared with MCF7-shCON tumors (Figures 7q and t). These data emphasize the crucial role of TAp63 in controlling the tumor-initiating potential of mammary cancer cells through regulation of LKB1 and the Hippo pathway.

### TAp63, components of cell polarity and the Hippo pathway are deregulated in human mammary adenocarcinoma

**Figure 8:**
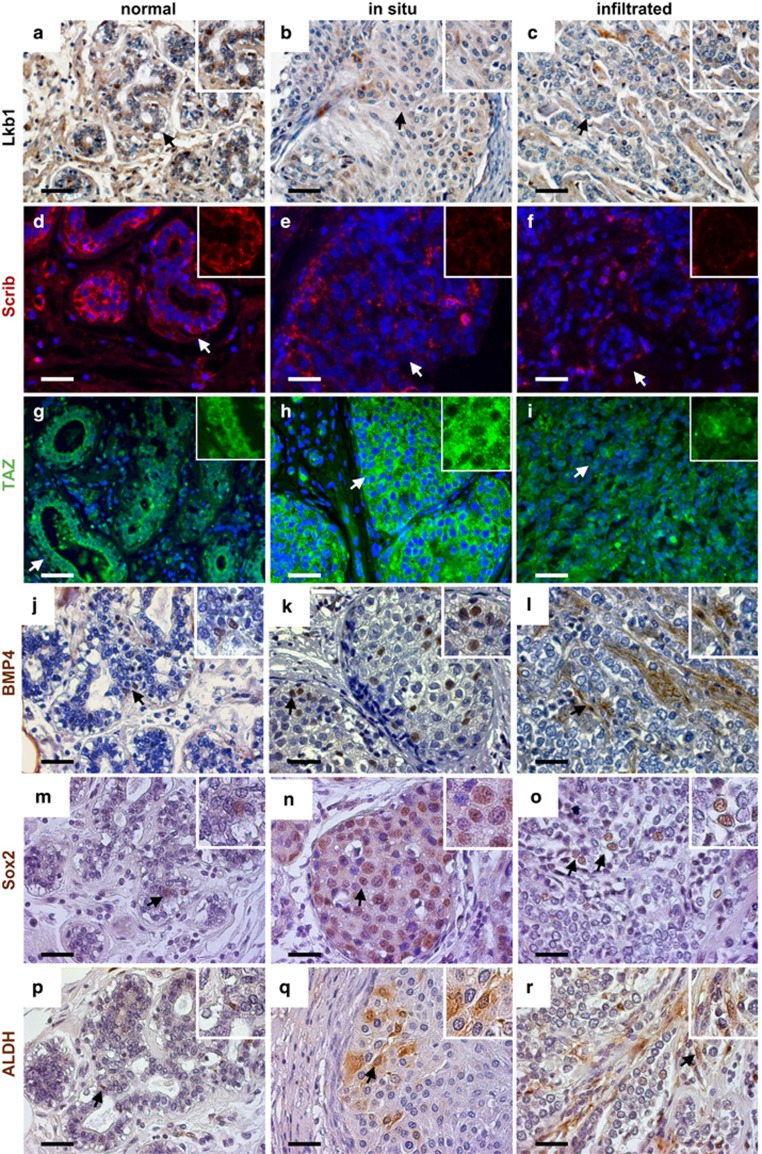
Components of cell polarity are deregulated in TAp63-deficient breast cancers. (**a**–**r**) Representative fluorescence or bright-field micrographs of normal human mammary tissue (normal), non-invasive mammary tumors (ductal carcinoma *in situ*) and invasive human mammary adenocarcinomas (infiltrated) using antibodies for LKB1 (brown) (**a**–**c**), Scrib (red) (**d**–**f**), TAZ (green) (**g**–**i**), BMP4 (brown) (**j**–**l**), Sox2 (brown) (**m**–**o**) and ALDH (brown) (**p**–**r**). DAPI (blue) or hematoxylin (purple) were used as counterstains. Arrows indicate areas further magnified in insets in the upper right corner of each panel. All scale bars represent 50 μm. Normal tissue *n*=6, *in situ* tissue *n*=20, infiltrated tissue *n*=12.

Our data indicate that TAp63 transcriptionally activates *LKB1*, which in turn regulates Scrib and the Hippo pathway through TAZ. To ask whether this pathway is deregulated in human mammary cancer through loss of TAp63, we tested 48 human breast samples, including normal, ductal carcinoma *in situ* and infiltrative mammary adenocarcinoma. We found that TAp63 is downregulated along with LKB1 and Scrib in ductal carcinoma *in situ* and infiltrative mammary adenocarcinomas (Figures 8a–f and Supplementary Table S4).^14^ We also found that TAZ is overexpressed in these samples along with its downstream target BMP4 (Figures 8g–l and Supplementary Table S4), indicating activation of the Hippo pathway in tumors deficient for TAp63. We next asked whether activation of the Hippo pathway through TAZ led to an increase in breast CSCs. Indeed, Sox2 and ALDH are expressed in these samples (Figures 8m–r and Supplementary Table S4). Taken together, these data further indicate that the Hippo pathway is activated through the TAp63 pathway and that this leads to an increase in TICs and CSCs through deregulation of cell polarity in the mammary gland.

## Discussion

p63 is a crucial regulator of epithelial stem cells and is frequently used as a diagnostic marker for breast cancer.^2, 5, 6, 10^ The N-terminal isoforms of p63 maintain distinct stem cell compartments in the skin.^5, 11^ TAp63, containing an acidic transactivation domain, serves to maintain dermal stem cells critical for wound healing and hair regeneration known as skin-derived precursors in quiescence.^5, 41^ ΔNp63, the p63 isoforms lacking the N-terminal acidic transactivation domain, is highly expressed in basal cells within the epidermis and is needed for terminal differentiation of the stratified epidermis.^11, 12^ Although p63's function in progenitor and stem cells in the skin has been well studied, the mechanisms employed by p63 and its isoforms to regulate stem cell proliferation remain poorly understood. Moreover, the roles of p63 isoforms in mammary gland development and the regulation of progenitor and stem cells in the mammary gland remain poorly defined. Although the roles of ΔNp63 in the mammary gland have been thoroughly examined^3, 4^ and ΔNp63 is necessary to sustain the self-renewal of mammary CSC,^42^ TAp63 has been ignored owing to its low level of expression in spite of its crucial role as a suppressor of tumorigenesis and metastasis.^9, 14, 15, 16, 43^ Here we used a *TAp63*-deficient mouse model to probe the function of TAp63 in the mammary gland and found that TAp63 regulates mammary TICs through the Hippo pathway. We found an accumulation of early TICs in transplanted *TAp63*-deficient mammary glands, in two orthotopic xenograft mammary cancer mouse models and in *TAp63−/−* mammary adenocarcinomas from mice and humans. We found that EMT occurs in the absence of *TAp63* leading to the acquisition of stem cell properties in MECs and cancer cells. These data provide a novel mechanism for TAp63's ability to suppress mammary adenocarcinoma through regulation of stem cells in the mammary gland.

The mammary gland is composed of basal and luminal cells. The various types of breast cancer are thought to arise from stem cells within the basal and luminal layers of the mammary gland. Luminal progenitor cells are thought to give rise to the basal-like subtype of breast cancer and MaSCs give rise to the claudin-low subtype of breast cancer.^44, 45, 46^ The mechanisms employed to maintain these stem cells populations in the mammary gland and their deregulation in cancer are not well understood. p63 is known to be crucial for the development and differentiation of stratified epithelium.^1, 2, 11, 12^ Mice deficient for all isoforms of p63 lack stratified epidermis and mammary glands.^2^ Mice lacking *TAp63* have fragile skin, develop blisters, wounds that never heal and alopecia.^5^ The epidermal phenotypes of *TAp63−/−* are due to the transcriptional regulation of *p57*^*Kip2*^ by TAp63 to maintain skin-derived precursors, a stem cell found to be critical for wound healing and hair regeneration,^5, 41^ in quiescence. When TAp63 is lost, skin-derived precursor cells proliferate inappropriately and are prematurely depleted resulting in the phenotypes seen.^5^ In contrast, ΔNp63 was found to be crucial for terminal differentiation of basal keratinocytes and is necessary for the maintenance of basal cells in the epidermis.^11, 12^ Given that the p63 isoforms have crucial roles to maintain these epidermal and dermal adult stem in the skin, we hypothesized that TAp63 may be having similar roles in the mammary gland owing to its importance as a suppressor of tumorigenesis and metastasis.^9, 14, 15, 16, 43^ The ΔNp63 is highly expressed in mammary epithelial basal cells and its role in maintaining MaSCs has recently been well described using a ΔNp63-deficient mouse model through regulation of Wnt signaling^3^; however, in these studies, the role of TAp63 could not be examined and was exclusively focused on the use of ΔNp63-deficient mouse models. Additionally, it is important to note that p63 is used as a diagnostic marker in breast cancer with no regard for the existence of distinct isoforms and their functions potentially leading to improper diagnosis and treatment of breast cancer patients. Here we used the tumor-prone *TAp63−/−* mouse model to specifically dissect the role of the TAp63 isoforms in the mammary gland. Consistent with previous reports,^3^ we found that lack of *TAp63* does not dramatically affect the developing mammary gland, as mammary glands from virgin *TAp63−/−* females have a higher proliferative index than WT mammary glands but are otherwise indistinguishable from those of WT females. However, we unveiled striking phenotypes subsequent to serial mammary transplantation, suggesting that TAp63 has similar roles in the mammary gland as it does in the skin, that is, TAp63 is activated in response to stress and is critical to maintain stem and progenitor cells in quiescence. Indeed, loss of *TAp63* led to an increase in TICs through the regulation of cell polarity and EMT. Moreover, mouse and mammary adenocarcinomas deficient for TAp63 and an orthotopic xenograft mouse model of mammary adenocarcinoma had an increased number of TICs, indicating that TAp63 serves to regulate stem cell properties and its loss leads to an expansion of TICs.

We previously reported that 8% of *TAp63−/−* mice spontaneously develop mammary adenocarcinoma late in life (9–16 months of age).^14^ In this study, *TAp63−/−* MECs were serially transplanted nine times in 10-week-old WT recipients. We did not observe any frank mammary gland adenocarcinomas but did detect *in situ* ductal carcinoma in the transplantation model. The difference in the models is most likely due to age and tumor latency. In fact, we did see mammary adenocarcinoma formation in MCF10-shTAp63 cells injected to SCID mice after 8 months (Figure 7e).

The Hippo pathway known to regulate organ size in *Drosophila* has been found to have important roles in the regulation of cell proliferation, cell polarity, stem cell maintenance and cancer in mammals.^22, 24, 25, 38^ The upstream regulators of the Hippo pathway are an area of intense investigation and tumor suppressors that may regulate this pathway are still being unveiled. We found that the tumor-suppressor TAp63 regulates the Hippo pathway effector TAZ through transcriptional regulation of *LKB1*, which has previously been shown to have important roles in stem cell regulation and metastasis.^22, 38, 47^ Our laboratory had already made two key discoveries: (1) TAp63 transcriptionally activates *Lkb1* in liver, muscle and fat tissues to regulate glucose metabolism,^40^ and (2) loss of *TAp63* results in the development of metastatic mammary adenocarcinoma.^14^ What was unknown were the mechanisms employed by TAp63 that lead to the development of mammary adenocarcinomas. Here we show that TAp63 also transcriptionally regulates *LKB1* in MECs and mammary cancer cell lines. Loss of regulation of LKB1 in *TAp63*-deficient MECs resulted in loss of Scrib expression and activation of the Hippo pathway through TAZ and a subsequent loss of cell polarity and accumulation of TICs. Importantly, re-expression of LKB1 in MECs lacking *TAp63* resulted in rescue of the aforementioned phenotypes. Cell polarity was restored and the number of MaSCs was reduced to WT levels. Taken together, we have unveiled mechanisms employed by the tumor- and metastasis-suppressor TAp63 to regulate stem cell properties in MECs, leading to the formation of TICs through regulation of the Hippo pathway. These observations are important for the appropriate use of p63 as a diagnostic marker and the potential treatments chosen for these patients.

## Materials and methods

### Animal studies and mammary gland transplantation

*TAp63−/−* and WT female mice on an enriched C57BL/6 background (95%) were used in this study. For mammary gland transplantation, the endogenous mammary epithelium was surgically removed from the four inguinal glands of WT females at 3 weeks of age to provide a cleared mammary fat pad.^48^ The donor mice were prepared as follows: fragments of 10-week-old WT or TAp63*−/−* mammary glands were inserted into the cleared mammary fat pads. WT was inserted on the right and *TAp63−/−* on the left. Ten weeks after mammary gland transplantation, fat pads were excised for histological and IHC analysis. All *in vivo* mouse procedures were approved by the IACUC at the University of Texas MD Anderson Cancer Center.

### Mammary gland limiting dilution assay

Primary mammary gland epithelial cells were isolated from 10-week-old WT and *TAp63−/−* female mice and injected into cleared mammary fat pads of WT female mice. Transplantation was performed using 2500, 1000, 500 and 250 of MECs of each genotype resuspended in 50% Matrigel and 50% phosphate-buffered saline (PBS).^48^ WT cells were injected on the right and *TAp63−/−* cells were injected on the left. The outgrowths of mammary glands were analyzed at 10 weeks posttransplantation by histology and IHC.

### Whole-mount mammary glands analyses

Mammary glands were removed from female mice, spread flat and fixed in 4% paraformaldehyde for 2 h at 4 °C. The glands were washed in 70% ethanol for 15 min followed by a brief rinse in PBS. The tissues were then stained in carmine alum solution (1 g of carmine and 2.5 g of aluminum potassium sulfate/500 ml of water) at room temperature overnight. The glands were flattened between glass slides to capture whole-mount images (× 10 magnification) using a Zeiss Stemi 200-CS microscope (Jena, Germany).

### Orthotopic xenograft mouse model

Female SCID mice (6 weeks old) were randomized into four groups: MCF10A-shCON (*n*=9), MCF10A-shTAp63 (*n*=12), MCF7-shCON (*n*=12), and MCF7-shTAp63 (*n*=12). All mice for MCF7 cells were treated by subcutaneous implantation of 17β-estradiol (0.72 mg 17β-estradiol, 90-day release, Innovative Research of America, Sarasota, FL, USA). MCF10A or MCF7 expressing a scrambled shRNA (shCON) or shTAp63 (1 × 10^6^ cells for MCF10A and 100 000 cells for MCF7 in matrigel) were implanted orthotopically into the mammary fat pads of the mice. Tumor xenografts were collected at 8 weeks after injection for MCF7 tumors and 8 months after injection for MCF10A tumors, photographed and analyzed using IHC. Total RNA was isolated from tumor xenografts for RT-PCR analysis.

### Cell lines and culture conditions

MCF10A cells were cultured in Dulbecco's modified Eagle's medium (DMEM)/F12 (1:1) media containing 5% horse serum, 10 μg/ml insulin, 20 ng/nl epidermal growth factor, 500 ng/ml hydrocortisone and primocin 100 μg/ml. Mouse epithelial cell lines were developed from mammary glands of WT and *TAp63−/−* mice at 10 weeks of age. Freshly dissected mammary glands were enzymatic dissociated overnight in DMEM/F12 medium containing collagenase (1 mg/ml), hyaluronidase (100 U/ml) and antibiotic–antimycotic solution (10 000 units/ml of penicillin, 10 000 μg/ml of streptomycin and 25 μg/ml of fungizone antimycotic). Cell pellets were washed two times with PBS+5% fetal bovine serum and were digested by 0.25% Trypsin–2.21 mM EDTA to get single cells. Trypsin was inactivated with PBS+5% fetal bovine serum. Cells were filtered through a 40 μm strainer to yield single-cell suspension. Cells were cultured in DMEM/F12 (1:1) media containing the same components used for MCF10A cells. All cultured cells were mycoplasma negative.

### *In vitro* lentivirus and adenovirus infections

shTAp63 and control lentivirus vectors were a gift from Leif Ellisen.^4^ MCF10A cells were infected with virus-containing media supplemented with 2 μg/ml polybrene for 24 h and were selected by puromycin for 3 days as described previously.^14^ Suspension of MCF10-shCON and MCF10A-shTAp63 cells were infected with human adenoviruses (Ad-LKB1) (Vector BioLabs, Malvern, PA, USA) or Ad-GFP (Vector Development Lab, Houston, TX, USA) for 1 h at a multiplicity of infection of 50. The efficiency of infection was quantified by assessing green fluorescent protein (GFP)-positive cells.

### Soft agar assay

MCF7 cells expressing a scrambled shRNA (shCON) or an shRNA for TAp63 (shTAp63) (50 000 cells) were seeded on the top layer in 0.3% agar in DMEM media in six-well plates. The base layer was composed of 1.5 ml of 0.6% agar in DMEM. Plates were allowed to solidify, then 1.5 ml DMEM was added to each well. Plates were incubated at 37 °C, and the media was changed every 3 days. Colonies were counted and photomicrographs were taken at day 21 using a Celigo instrument (Nexcelom, Lawrence, MA, USA).

### Western blotting analysis

Western blotting analysis was performed as described previously.^5^ Primary antibodies used were as follows: slug (Santa Cruz, 1:1000; Santa Cruz, CA, USA), sox9 (Millipore, 1:2000; Darmstadt, Germany), ALDH1 (BD Bioscience, 1:500; San Jose, CA, USA), αSMA (Sigma, 1:1000; St Louis, MO, USA), LKB1 (Cell Signaling, 1:500; Beverly, MA, USA), Scrib (Santa Cruz, 1:1000), YAP/TAZ (Cell Signaling, 1:500), CTGF (Santa Cruz, 1:500), TAp63 (BioLegend, 1:500; San Diego, CA, USA), ΔNp63 (BioLegend, 1:500), BMP4 (Abcam, 1:20000; Cambridge, MA, USA), E-cadherin (Cell Signaling, 1:2000), and sox2 (Abcam, 1:1000). Corresponding secondary antibodies conjugated to horseradish peroxidase (Amersham Biosciences, 1:5000; Marlborough, MA, USA) were used. Detection was performed using the ECL Plus Kit (Amersham). Actin was used as a loading control.

### Immunofluorescence and immunohistochemistry

For IF, paraffin-embedded sections were stained as described previously^40^ using the following primary antibodies: TAZ (Cell Signaling, 1:1000), K18 (Sigma, 1:200), αSMA (Sigma, 1:250), Ki67 (Abcam, 1:1000), caspase 3 (Cell Signaling, 1:50), AQP (Calbiochem, 1:100; Darmstadt, Germany), Scrib (Santa Cruz, 1:100), Vangl2 (Santa Cruz, 1:50), GM130 (BD Bioscience, 1:150), Sox9 (Millipore, 1:100), NKCC and Slug (Santa Cruz, 1:100). Alexa 488 and Alex 568-conjugated secondary antibodies (ThermoFisher Scientific, Molecular Probes, Waltham, MA, USA) were used at 1:1000. DAPI (6-diamidino-2-phenylindole) was used to counterstain nuclei. Images were acquired using Zeiss Axiovert 40CFL microscope. For IHC, the following primary antibodies were used: vimentin (BD Bioscience, 1:200), LKB1 (Cell Signaling, 1:100), BMP4 (Abcam, 1:100), sox2 (Abcam, 1:100), and ALDH (BD Bioscience, 1:50). For detection, the ImmPRESS REAGENT KIT (Vector Laboratories, MP-7500) was used followed by the DAB kit (Vector Laboratories, SK-4100) and counterstained with hematoxylin (Vector Laboratories, H-3041).

### Chromatin immunoprecipitation

Mouse epithelial cells of the indicated genotypes were cultured as previously described.^5^ Similarly, human MCF10A cells treated with a scrambled non-targeting (NT) shRNA or shTAp63 were grown to near confluence. TAp63 chromatin immunoprecipitation analysis was performed using anti-TAp63 antibody (D20, SantaCruz, 2 μg/mg protein) or immunoglobulin G as described previously.^40^ Mouse TAp63-binding sites on LKB1 promoter were validated with the primers as described previously.^40^ Putative human TAp63-binding sites were found by scanning 5000 bp upstream of the 5′ untranslated region and intron 1 of the *LKB1* gene utilizing the Genomatix software prediction tool (Ann Arbor, MI, USA). qRT-PCR was performed using primers in the human LKB1 promoter: (1) Site 1 (−3169)—forward 5′-CCTCCCAAAGTGCTGGGATTA-3′ and (−3068)—reverse 5′-CTGGGATTACAGGCATGAGG-3′, (2) Intron 1: Site 2 (+6548)—forward 5′-ATGTTGTCCAGGCTGGTCTC-3′ and (+6635)—reverse 5′-GTGAACGTGGCGAAGCAGT-3′, and (3) non-specific: (−221)—forward 5′-ATGGCAGGTTCAACCAACG-3′ and (−122)—reverse 5′-GCCGCCATCTTGTTTACCT-3′.

### Fluorescence-activated cell sorting (FACS) and flow cytometry

MCF10A-shCON and -shTAp63 cells were labeled with CD24-PE (Biolegend, 1:00) and CD44-Alexa 488 (Biolegend, 1:00) at a concentration of 10 million cells/ml for 20 min and were subjected to FACS analysis and sorting on a triple laser MoFlo FACs machine (Cytomation, Fort Collins, CO, USA). Data analysis was performed using the FlowJo software (Ashland, OR, USA).

### Mammary epithelial 3D cell culture

Mammary epithelial 3D cell culture (3D culture) was performed as described previously in growth factor-reduced Matrigel (BD Biosciences).^49^ For MCF10A cell-infected derivative cell lines, 5000 cells were seeded in each well of an eight-well chamber slide. WT and *TAp63−/−* primary mouse MECs were isolated from mammary organoids.^50, 51^ Briefly, mammary glands were removed from 8- to 12-week-old female mice and minced 40–50 times with a scalpel. The epithelial compartment was separated from the stromal tissues using collagenase in PBS (2 mg/ml) digestion. Differential centrifugation was used to separate epithelial organoids from single cells. Pellets containing the mammary organoids were digested using 0.05% Trypsin–2.21 mM EDTA to obtain single cells. In all, 10 000 cells were seeded per well of an eight-well chamber slide.

### *In vitro* wound healing assay

Confluent MCF10A cell monolayers were treated with 4 μg/ml mitomycin C for 2 h to stop cell proliferation. The monolayer of cells was then ‘wounded' by scratching the surface with a P200 micropipette tip. Six hours following the wound, cells were fixed in 4% paraformaldehyde for immunofluorescent staining. Images were captured using an inverted Zeiss Axiovert 40CFL microscope. For time-lapse imaging, the cells were labeled with nuclear stain (Hoechst, no. 33342, 1 μg/ml) for 20 min. Time-lapse imaging was performed for 24 h on an inverted Nikon Ti microscope (Melville, NY, USA) equipped with phase-contrast optics and a Hamamatsu Flash 4.0 camera (Hamamatsu, Japan). Nikon elements software was used for acquisition and analysis of the time-lapse data.

### RNA-Seq and bioinformatics analysis

RNA-sequencing of WT and *TAp63−/−* mouse epithelial cells was performed as described previously.^8, 11^ ESC, hNMSC and Hippo and Yap-conserved pathway signatures from previously published studies (Supplementary Table S2)^22, 28, 34, 35^ were compared with *TAp63−/−* and WT MEC signatures using Gene Set Enrichment Analysis from the Broad Institute (Cambridge, MA, USA). Gene Set Enrichment Analysis settings were 1000 permutations, classic scoring scheme and maximum probe mode. A positive and negative normalized enrichment score indicate same and opposite direction of signature, respectively. In pathway analysis, we utilized BP, KEGG and Reactome sources in order to identify pathways enriched in our signatures. *P*-value was set at <0.05 for significance.

### Statistical analysis

All data are represented as mean±s.e.m. One-way analysis of variance or Student's *t*-test was used for statistical comparison between two groups. A *P*-value of 0.05 was considered significant. Sample sizes were six for all *in vivo* mouse experiments and at least in triplicate for all cell culture assays.
