# Supplementary material for: TAp63 suppresses mammary tumorigenesis through regulation of the Hippo pathway
Source: Oncogene. 2016 Nov 21;36(17):2377–93. doi: 10.1038/onc.2016.388 (PMC5415945; doi:10.1038/onc.2016.388)
Supplement: Supplementary Figures and Tables [file onc2016388x1.pdf]

**Supplementary Figure 1**

| | WT | | TAp63-/- | | |
|---|---|---|---|---|---|
| Transplant Generation (TG) | Outgrowth rate | Percent Fat Pad Filled | Outgrowth rate | Percent Fat Pad Filled | P value % Filled |
| TG1 | 18/18<br>(100%) | ● X18 | 18/18<br>(100%) | ● X18 | |
| TG2 | 18/18<br>(100%) | ● X18 | 18/18<br>(100%) | ● X18 | |
| TG3 | 15/18<br>(83%) | ● X12<br>◕ X3<br>○ X3 | 6/18<br>(50%) | ● X8<br>◕ X4<br>○ X3 | 0.3151 |
| TG4 | 14/23<br>(61%) | ● X5<br>◕ X7<br>◔ X2<br>○ X9 | 19/23<br>(83%) | ● X8<br>◕ X9<br>◔ X2<br>○ X4 | 0.1230 |
| TG5 | 7/12<br>(58%) | ◕ X2<br>◔ X5<br>○ X5 | 10/12<br>(83%) | ● X2<br>◕ X5<br>◔ X3<br>○ X2 | 0.0154* |
| TG6 | 0/12<br>(0%) | ○ X12 | 7/12<br>(58%) | ● X1<br>◕ X3<br>◔ X3<br>○ X5 | 0.0151* |
| TG7 | | | 5/14<br>(36%) | ● X1<br>◕ X1<br>◔ X3<br>○ X9 | |
| TG8 | | | 5/8<br>(62%) | ◕ X1<br>◔ X4<br>○ X3 | |

Supplementary Figure 2

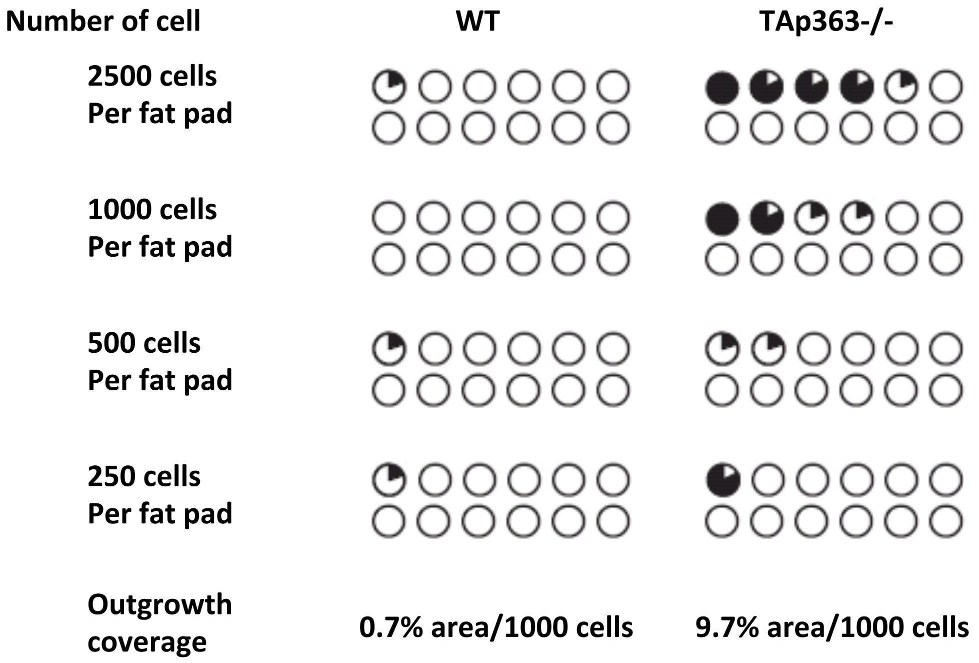

**Supplementary Figure 3**

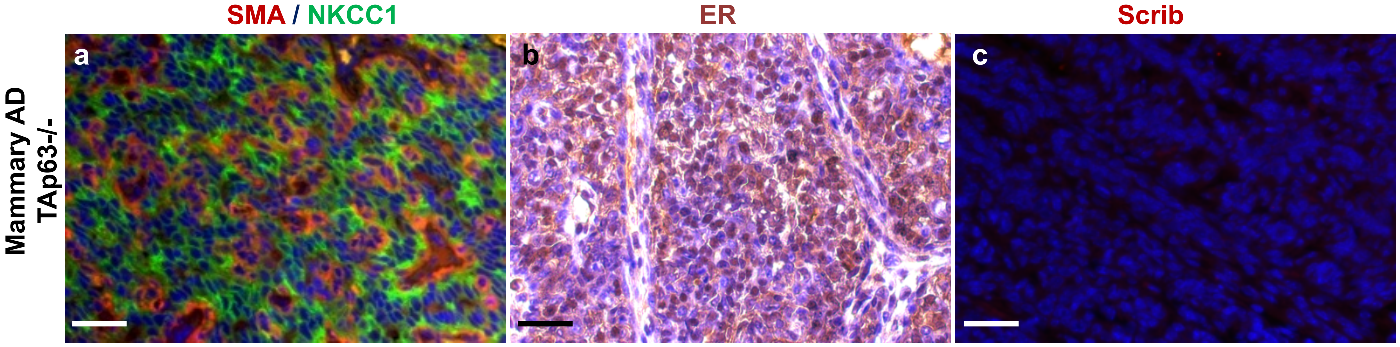

## Supplementary Figure 4

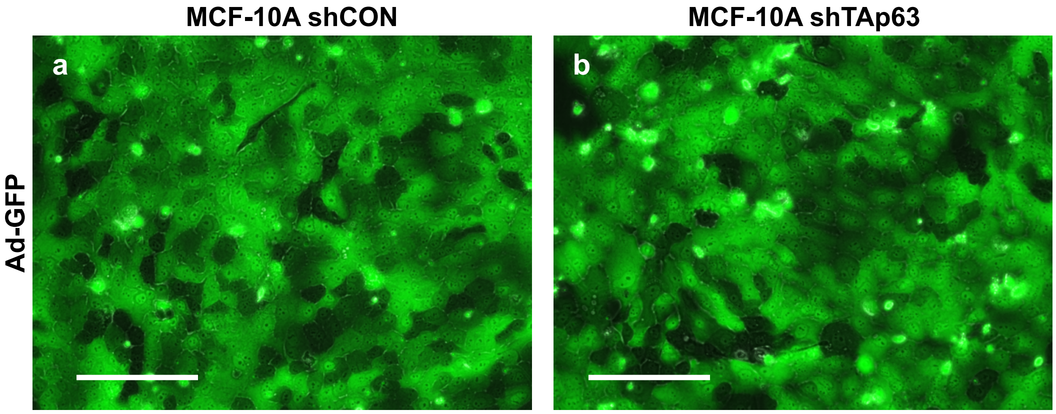

Supplementary Figure 5

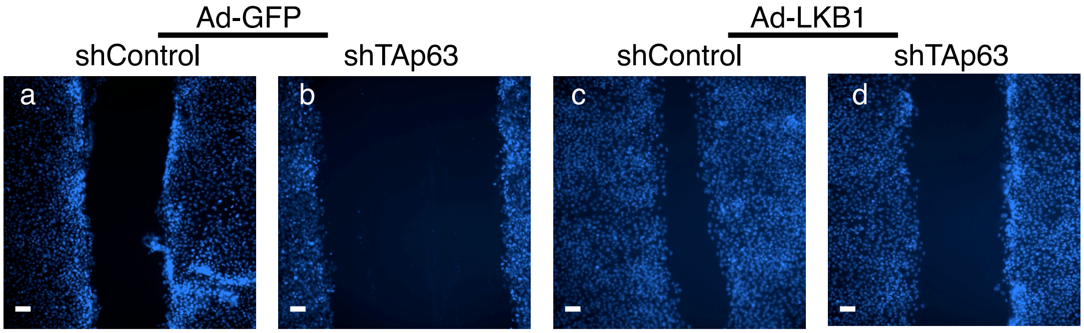

Supplementary Figure 6

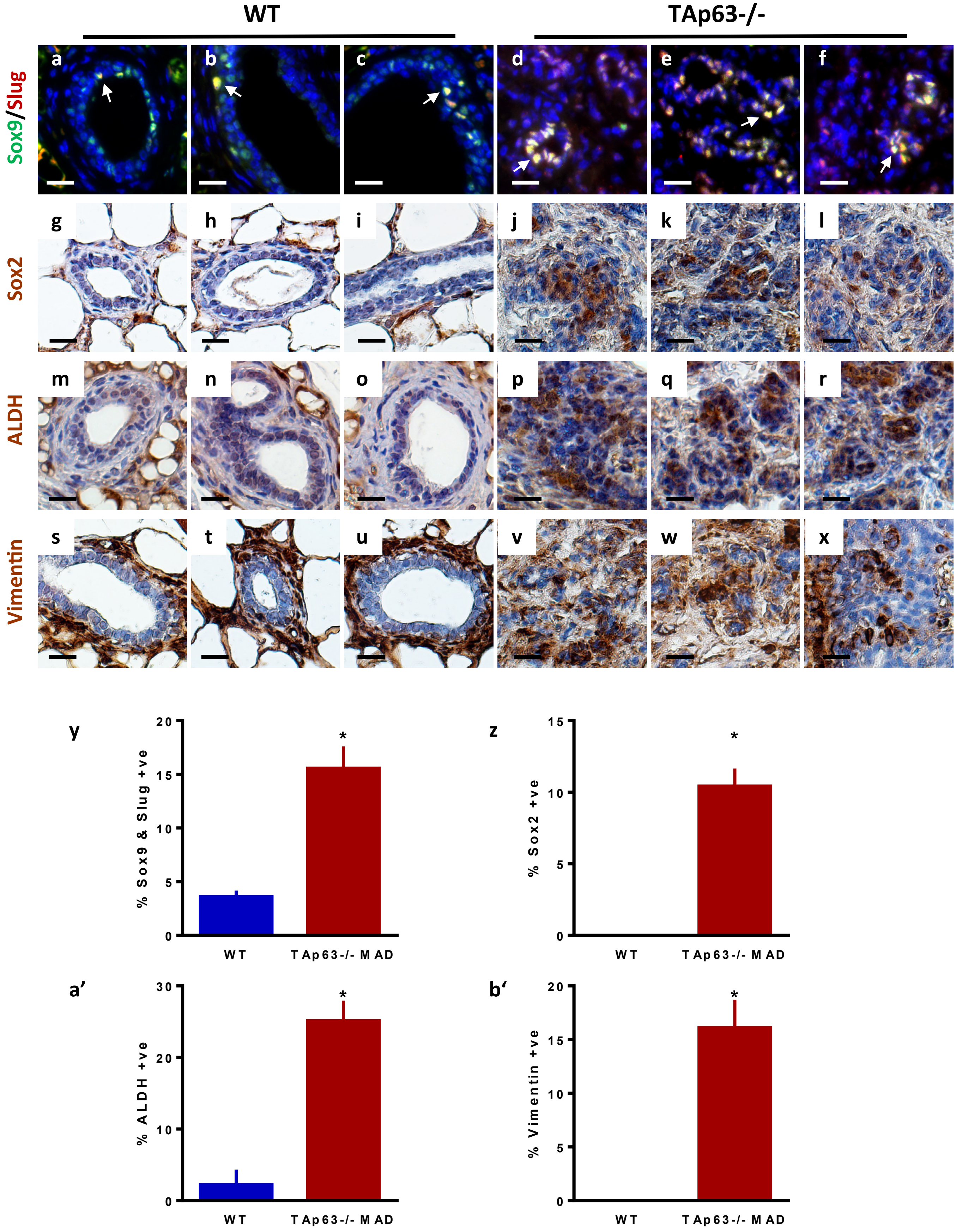

**Supplementary Table 1. Summary of out growth coverage of fat pad repopulation (%) following serial transplantation**

| % of fat pad | TG1 | TG2 | TG3 | TG4 | TG5 | TG6 | TG7 | TG8 | TG9 |
|---|---|---|---|---|---|---|---|---|---|
| **WT** | 100±0 | 100±0 | 74±39 | 41±42 | 10±50 | 0 | 0 | 0 | 0 |
| **TAp63-/-** | 100±23 | 94±24 | 61±39 | 59±36 | 38±34 | 25±32 | 12±27 | 8±11 | 0 |

**TG=transplanted generation**
**Number indicates percentage of mammary gland repopulation ± standard error of the mean**

**Supplementary Table 2. *TAp63-/-* mammary epithelial cell (MECs) are significantly enriched in both embryonic and normal human mammary stem cell signatures**

| Signature | Q-value | Normalized Enrichment Score (NES) | Reference |
|---|---|---|---|
| hNMSC (Up) | <0.0001 | +2.68 | Pece et al., *Cell* 2010 |
| hNMSC (Down) | <0.0001 | -2.32 | Pece et al., *Cell* 2010 |
| Embryonic stem cells 1 (Up) | <0.0001 | +3.68 | Ben-Porath et al., *Nature Genetics* 2008 |
| Embryonic Stem cells 2 (Up) | 0.325 | +1.09 | Ben-Porath et al., *Nature Genetics* 2008 |
| Yap Conserved (Up) | 0.4 | -0.97 | Cordesoni et al., *Cell* 2011 |
| Hippo (Up) | 0.204 | -1.23 | Mohseni et al., *Nature Cell Biology* 2014 |

**Supplementary Table 3. TAp63 binding sites on *LKB1* promoter and intron 1**

| Element | Location | Sequence | MM/Spacer |
|---|---|---|---|
| LKB1-promoter | -3078 to -3058 | aggCATGgtgcctCATGcct | 5/0 |
| LKB1-intron1 | +5278 to +5304 | actCAAGctatccgcctgcCTGGgcc | 6/6 |
| Non-specific | -220 to -189 | tggCAGGttcaaccaacgggtgggCACGtcg | 2/11 |

Mismatches are shown in red text.
**MM**: the number of mismatches in each binding site.
**Spacer**: number of nucleotides between two half sites.

**Supplementary Table 4. Summary of Scrib staining in human breast cancer tissues**

| Scrib | J | J+C | C | negative | N+C |
|---|---|---|---|---|---|
| Normal (4) | 3 (75%) | 1 (25%) | 0 | 0 | 0 |
| Hyperplasia (10) | 1 (10%) | 3 (30%) | c | 3 (30%) | 0 |
| Malignant (33) | 4 (12%) | 10 (30%) | 14 (42%) | 3 (9%) | 2 (6%) |

J=cell junction, C=cytoplasm, N=nucleus, negative = no expression

**Supplementary Table 5. Summary of TAZ staining in human breast cancer tissues**

| **TAZ** | **negative** | **+** | **++** | **+++** |
|---|---|---|---|---|
| Normal (4) | 0 | 3 (75%) | 1 (25%) | 0 |
| Hyperplasia (10) | 0 | 3 (30%) | 2 (30%) | 5 (50%) |
| Malignant (33) | 18 (55%) | 6 (18%) | 5 (15%) | 4 (12%) |

negative = no expression, + indicates level of positive expression

**Supplementary Table 6. Summary of LKB1 staining in human breast cancer tissues**

| LKB1 | cytoplasm | | | nuclear | | |
|---|---|---|---|---|---|---|
| | **negative** | **+** | **>++** | **negative** | **+** | **>++** |
| Normal (3) | 0 | 3 (100%) | 0 | 0 | 3 (100%) | 0 |
| Hyperplasia (10) | 1 (10%) | 9 (90%) | 0 | 6 (60%) | 4 (40%) | 0 |
| Malignant (20) | 10 (50%)) | 7 (35%) | 3 (15%) | 20 (100%) | 0 | 0 |

negative = no expression, + indicates level of positive expression

**Supplementary Table 7. Summary of BMP4 staining in human breast cancer tissues**

| **BMP4** | **negative** | **+** | **++** |
|---|---|---|---|
| Normal (3) | 0 | 3 (100%) | 0 |
| Hyperplasia (9) | 0 | 1 (31%) | 8 (89%) |
| Malignant (19) | 3 (16%) | 6 (32%) | 10 (52%) |

negative = no expression, + indicates level of positive expression

**Supplementary Table 8. Summary of Sox2 staining in human breast cancer tissues**

| **Sox2** | **negative** | **+** | **>++** |
|---|---|---|---|
| Normal (4) | 3 (75%) | 1 (25%) | 0 |
| Hyperplasia (9) | 1 (31%) | 1 (31%) | 7 (78%) |
| Malignant (32) | 6 (19%) | 10 (31%) | 16 (50%) |

negative = no expression, + indicates level of positive expression

**Supplementary Table 9. Summary of ALDH staining in human breast cancer tissues**

| ALDH | negative | <++ | >++ |
|---|---|---|---|
| Normal (3) | 1 (33%) | 2 (66%) | 0 |
| Hyperplasia (9) | 3 (30%) | 4 (44%) | 2 (22%) |
| Malignant (29) | 1 (3%) | 19 (66%) | 9 (31%) |

negative = no expression, + indicates level of positive expression
