# Supplementary material for: TAp63 suppresses mammary tumorigenesis through regulation of the Hippo pathway
Source: Oncogene. 2016 Nov 21;36(17):2377–93. doi: 10.1038/onc.2016.388 (PMC5415945; doi:10.1038/onc.2016.388)
Supplement: Supplementary Figure Legends [file onc2016388x2.pdf]

Supplemental Figure Legends

Supplementary Figure 1. ***TAp63-/- mammary glands have increased regenerative potential.*** Pie graphs indicate the outgrowth coverage of mammary gland fat pads after serial transplantation. Each pie graph represents one mammary gland and the blackened area represents the percentage of the fat pad filled with mammary gland outgrowth. "X" followed by a number indicate the number of mammary fat pads examined for each genotype and transplantation. = indicates 80–100%, = indicates 30–80%, = indicates 0–30%, and = indicates no reconstitution. Asterisk indicates significant difference.

Supplementary Figure 2. ***TAp63-/- mammary glands have an increase in tumor initiating cells, TICs, as assessed by limiting dilution assay***. Pie graphs indicate the outgrowth coverage of mammary gland fat pads after limiting dilution assay. Each pie graph represents one mammary gland and the blackened area represents the percentage of the fat pad filled with mammary gland outgrowth. = indicates 80–100%, = indicates 30–80%, = indicates 0–30%, and = indicates no reconstitution.

Supplementary Figure 3. ***TAp63-/- mice develop ER positive luminal mammary gland tumors.*** **(a-c)** Representative brightfield or fluorescent micrographs of mammary adenocarcinomas from *TAp63-/-* mice and immunostained using antibodies for smooth muscle actin (SMA) (red) and Na-Cl-K co-transporter (NKCC) (green) **(a)**, estrogen receptor (ER) (brown) **(b),** and scribble (Scrib) (red) **(c)**. DAPI (blue) or hematoxylin (purple) were used as counterstains. Scale bars represent 50μm.

Supplementary Figure 4. ***Infection efficiency of MCF10A cells transduced with adenoviruses as assessed by GFP.*** **(a & b)** Adenovirus infection rate is greater than 90% as indicated by GFP expression in MCF10A-shCON **(a)** and MCF10A-shTAp63 **(b)** cells transduced with adenovirus-GFP (Ad-GFP). Scale bars represent 50μm.

Supplementary Figure 5. ***LKB1 rescues cell migration and cell polarity defects in MCF10A cells deficient for TAp63.*** **(a-d)** Live imaging microvideography using fluorescent time-lapse microscopy of wound healing in a 24 hour time frame of MCF10A cells expressing a non-specific control shRNA (shCON) transduced with an empty adenovirus (Ad-GFP) **(a)** or

MCF10A cells expressing a TAp63 specific shRNA (shTAp63) and transduced with Ad-GFP **(b)**. Live imaging movies of wound healing in MCF10A-shCON cells transduced with adenovirus-LKB1 (Ad-LKB1) **(c)** or MCF10A-shTAp63 cells transduced with Ad-LKB1 **(d).** Video Still snapshot taken at 12 hours. Scale bars represent 50μm.

Supplementary Figure 6. ***TAp63-/- mammary glands have increased numbers of cancer stem cells, CSC.*** (**a-x**) IF or IHC staining of mammary adenocarcinomas from *TAp63-/-* mice (**d-f, j-i, p-r, v-x**) or MG from 5 week old WT mice (**a-c, g-I, m-o, s-u**) using antibodies for Sox9 (green), slug (red), Sox2 (brown), ALDH (brown), vimentin (brown) and E-cadherin (green). DAPI (blue) or hematoxylin (purple) were used as counterstains. Arrow indicates example of double positive nuclei (yellow). All scale bars represent 50μm. n=6 for all experiments. (**y-b'**) Bar graph showing quantification of Sox9 and Slug double positive cells, Sox2, ALDH and Vimentin positive cells from panel's a - x. MAD: Mammary Adenocarcinoma.
